# An array of highly flexible electrodes with a tailored configuration locked by gelatin during implantation—initial evaluation in cortex cerebri of awake rats

**DOI:** 10.3389/fnins.2015.00331

**Published:** 2015-09-25

**Authors:** Johan Agorelius, Fotios Tsanakalis, Annika Friberg, Palmi T. Thorbergsson, Lina M. E. Pettersson, Jens Schouenborg

**Affiliations:** ^1^Department of Experimental Medical Science, Neuronano Research Centre, Lund UniversityLund, Sweden; ^2^The Nanometer Structure Consortium, Lund UniversityLund, Sweden

**Keywords:** brain machine interface, flexible electronic implant, neural probe, embedding material, brain micro-motions, stable neural recordings, mechanical compliance, neuromodulation

## Abstract

**Background:** A major challenge in the field of neural interfaces is to overcome the problem of poor stability of neuronal recordings, which impedes long-term studies of individual neurons in the brain. Conceivably, unstable recordings reflect relative movements between electrode and tissue. To address this challenge, we have developed a new ultra-flexible electrode array and evaluated its performance in awake non-restrained animals.

**Methods:**An array of eight separated gold leads (4 × 10 μm), individually flexible in 3D, were cut from a gold sheet using laser milling and insulated with Parylene C. To provide structural support during implantation into rat cortex, the electrode array was embedded in a hard gelatin based material, which dissolves after implantation. Recordings were made during 3 weeks. At termination, the animals were perfused with fixative and frozen to prevent dislocation of the implanted electrodes. A thick slice of brain tissue, with the electrode array still in situ, was made transparent using methyl salicylate to evaluate the conformation of the implanted electrode array.

**Results:** Median noise levels and signal/noise remained relatively stable during the 3 week observation period; 4.3–5.9 μV and 2.8–4.2, respectively. The spike amplitudes were often quite stable within recording sessions and for 15% of recordings where single-units were identified, the highest-SNR unit had an amplitude higher than 150 μV. In addition, high correlations (>0.96) between unit waveforms recorded at different time points were obtained for 58% of the electrode sites. The structure of the electrode array was well preserved 3 weeks after implantation.

**Conclusions:** A new implantable multichannel neural interface, comprising electrodes individually flexible in 3D that retain its architecture and functionality after implantation has been developed. Since the new neural interface design is adaptable, it offers a versatile tool to explore the function of various brain structures.

## Introduction

A major challenge in the field of Brain-Machine Interfaces (BMIs) is that the stability of neuronal recordings often is insufficient for studies of single neuronal activity over long periods of time. Unstable electrode position in the tissue can occur if the implant is too rigid to follow the movements of the brain during for example the respiratory and cardiac cycles (Britt and Rossi, 1982) but can also be caused by body movements (Jackson and Fetz, 2007; Santhanam et al., 2007). Moreover, the quality, i.e., signal-to-noise ratio, commonly gradually deteriorates over time (Rousche and Normann, 1998; Williams et al., 1999; Polikov et al., 2005). This deterioration may in part also be due to instability, since micro-motions between probe and tissue are likely to cause continuous irritation or indeed injury of the tissue that result in long-term glial activation and unfavorable effects on nearby neurons (Turner et al., 1999; Williams et al., 1999; Szarowski et al., 2003; Biran et al., 2005; Gilletti and Muthuswamy, 2006; Seymour and Kipke, 2007; Thelin et al., 2011; Lind et al., 2013). These glial reactions and effects on nearby neurons are known to impair recording quality (Nolta et al., 2015).

The largest micro-motions occur between the brain and the skull in the awake freely moving animals (Kim et al., 2004; Gilletti and Muthuswamy, 2006; Biran et al., 2007). For this reason, flexible leads on the cortical surface connecting the implanted rather stiff electrodes to the electronics have been developed (Rousche and Normann, 1998; Jackson and Fetz, 2007; Musallam et al., 2007). However, there are also significant micro-motions inside the brain caused by propagating waves of blood inside the vessels (Eide, 2008; Wagshul et al., 2011). The propagating nature of these movements implies that the movements inside the brain are not uniform. On top of these movements, there are movements caused by respiration. If the implanted electrodes cannot follow these tissue movements in all three dimensions, micro-forces/micro-movements between tissue and the recording sites on the electrodes will ensue. Indeed, it is a common experience, when performing acute electrophysiological experiments using stiff microelectrodes for extracellular recordings, that substantial pulsative movements inside the brain can occur as an effect of e.g., heart beats despite stabilization of the surface. As a result, such recordings tend to be short lived if the electrode is too close to the recorded neurons. Hence, to allow close contacts with neurons for longer periods of time, a high degree of mechanical compliance between electrodes and tissue is likely to be beneficial.

To improve mechanical compliance between electrodes and tissue, efforts have been made to increase the flexibility of the implanted electrodes themselves (Rousche et al., 2001; Kipke et al., 2002; Takeuchi et al., 2005; Stice et al., 2007; Bae et al., 2008; Krüger et al., 2010; Andrei et al., 2012; Felix et al., 2012; Lee et al., 2012; Kuo et al., 2013; Ejserholm et al., 2014; Kozai et al., 2014; Sohal et al., 2014). However, some of them are too flexible to be implanted on their own, and thus structural support in the form of a stiff guide or cannula (Kipke et al., 2002; Felix et al., 2012; Kuo et al., 2013; De Faveri et al., 2014; Sohal et al., 2014) has been used. These guides/cannulae cause additional stab wound-like injuries on their own, and on removal risk perturbing the position of the implanted flexible electrodes. Many of these flexible electrodes embed the conductive leads in a polymer sheet. However, this reduces their flexibility to one dimension only, and since the electrode leads are fixed in the polymer they cannot move independently of each other. In addition, such chip-like constructions also permanently split the tissue into two halves, causing an irreversible disruption of communication between neural networks on the two sides and therefore create a less than ideal situation when analyzing the function of neuronal networks. Hence, the challenge of providing an implantable array of independent highly flexible electrodes and that can be provided with an architecture adapted to the target tissue still remains.

The aim of the present project was thus to develop a fully functional and implantable array of ultra-thin electrodes, individually flexible in three dimensions after implantation. We present a new type of array of ultra-flexible electrodes which by being embedded in a hard but dissolvable gelatin based material retains its conformation and arrangement during implantation. Preliminary performance tests in awake rats showed encouraging results.

## Materials and methods

### Design and fabrication of the electrode array

**Figure 1:**
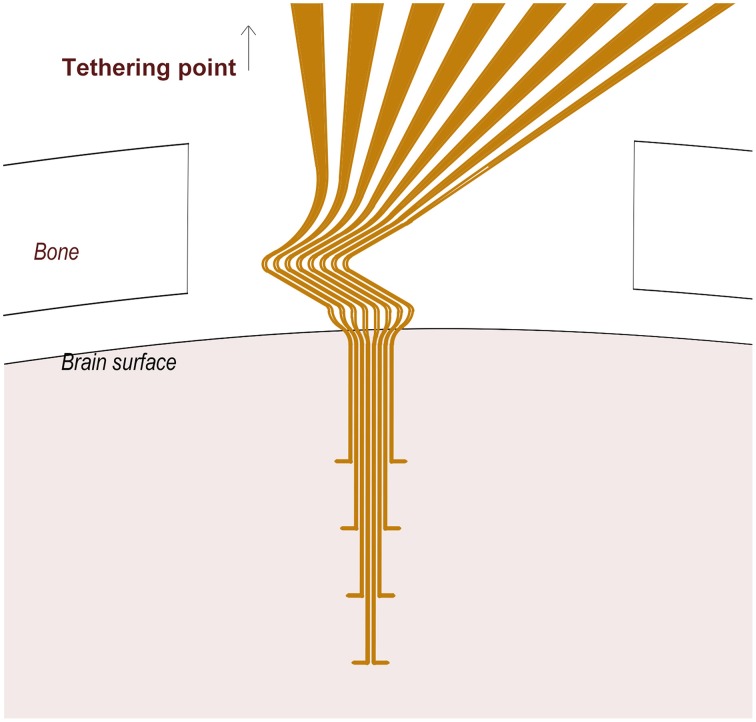
**A schematic of the 3D flexible electrode array**. Note the Z-shaped proximal part of the electrode array located between the cranium and the brain surface, and the tips (10 μm) of the distal protrusions which serve as recording sites.

We designed an array of eight wavy electrodes, thus flexible/extendable in three dimensions, each equipped with a distal protruding branch from where neuronal recordings could be made (Figure 1). The electrode array conformation was designed to allow recordings from different cortical laminae. The design was created using AutoCAD 2011 software and digitally imported into a Laser Machining System (Laser mill 50, standard micro-milling system, Nd:YAG laser system, New Wave Research, Inc., Fremont, CA). A 4 μm-thick sheet of gold foil (Rosenobel-Doppelgold 23 ¾ karat, J.G. Eytzinger GmbH, Germany) was fastened onto a microscope glass slide using polyethylene glycol (Sigma-Aldrich). Gold leads, 10 μm in width were milled under a fixed 20X objective using pulsed green laser light (wavelength 532 nm, energy density 10 J/cm^2^).

The glass slide containing the milled electrodes was mounted with silicon (Elastosil A07) and aligned on a metal frame to which a printed circuit board (PCB) was attached. The individual leads were manually soldered to the PCB one by one using a flux mixture solution (62% Sn, 36% Pb, 2% Ag) applied between the electrodes to be soldered and the contacts on the PCB. This process was performed under a stereomicroscope (SMZ1500, Nikon Instruments Europe BV, The Netherlands). To wash away the remaining flux mixture from between the PCB leads and to release the electrode leads from the glass slide, the metal frame was immersed in 70% ethanol, after which the glass slide was carefully removed manually.

An acrylic coating (HumiSeal advanced protective coating for electronics) was applied as protection over the soldered leads and over the bottom part of the PCB. The electrode array, including the PCB, was then coated with Parylene C (4 μm thick) using a Compact Bench Top Coating System (Labtop 3000, Para Tech Coating Inc., CA, US).

There were no obvious inherent strain forces in the construct since the gold electrodes were not displaced after being cut out from the gold sheet and also kept their conformation when insulated with parylene C.

To prepare the active recording sites on the protruding tips, the parylene coating was photo-ablated on both sides, over an area extending 10 μm from each electrode lead tip, using focused UV radiation (wavelength 355 nm, energy density 1.4 J/cm^2^) under a 20x objective. The successful exposure of the lead recording sites was monitored in real time during the ablation process and subsequently carefully confirmed with Scanning Electron Microscopy (SEM).

A ~40 mm long, 150 μm thick, un-insulated silver wire (Advent research Materials Ltd, England) was attached to the chip in the assigned “ground” position in order to be used as animal ground for the recordings. The electrode, connected to the chip, was finally released from the frame.

### Impedance spectroscopy

The electrical properties of each electrode lead were evaluated by impedance spectroscopy (Gamry Instruments, Series G 300, USA). After immersion in 0.9% saline solution, the impedance of the individual channels was measured by applying a 10 mV AC voltage with a frequency of 1 KHz, using a large Pt wire as counter electrode and an Ag/AgCl electrode in phosphate buffer as reference. The mean impedance of the individual channels was 832 kΩ (*SD* = 230 kΩ). It should be noted that repeated impedance measurements did not significantly change the impedance of the electrodes.

### Embedding of the electrodes

The gold electrodes used in this study are highly flexible, for instance they cannot be inserted into water without bending. To provide structural support during implantation, the ultra-flexible electrode array was embedded in a hard, but dissolvable, gelatin B based matrix material. The gelatin matrix was prepared by slowly heating a mixture of 3 g gelatin (Gelatin powder Ph.Eur., 24360.368, WWR International AB, Sweden), 750 mg PEG 400 (81172-1L, Sigma-Aldrich Chemie GmbH, Germany), 150 mg glycerol 98% (GRP Rectapur, 24 387.361, VWR, BDH Prolabo, France), and 7 mL Millipore water (Millipak-20 Filter Unit 0.22 μm 1/4 in), to 70°C, giving a final concentration of 27.5% gelatin, 1.4% glycerol and 6.9% PEG. The mixture was then placed on a hot plate while stirring. The electrode array was placed inside a custom made Poly Methyl Methacrylate (PMMA) mold and the gelatin mixture was injected. The humidity during drying was held at 21%, and the gelatin was dried first 24 h inside the mold, and then 24 h with the top lid of the mold removed. The remaining water content after drying was estimated to be below 4% (by measuring the weight of the probes (*n* = 5) before and after drying). This method enabled production of a probe embedded in a gelatin matrix with a sharp rigid tip that allowed for penetration of the brain tissue (**Figure 3B**).

Finally, a thin coat of a water retardation material was applied in order to delay dissolution of the matrix material until the implant was fully implanted. In short, the gelatin-embedded electrode array was dip-coated in a 5% (w/w) solution of kollicoat (Fluka, Sigma-Aldrich Sweden AB) in 99.7% ethanol, two times with a drying interval of 24 h in between each coat, and at least 1 h between the last coating and implantation.

### Animal surgery

Approval for the experiments was obtained in advance from the Malmö/Lund Animal Ethics Committee on Animal Experiments (ethical permit M60-13) and all experiments in this work conform to the regulatory standards of this approval. All rats had free access to food and water and were kept in a 12-h light/dark cycle at a constant environmental temperature of 21°C and 65% humidity.

Female Sprague-Dawley rats were implanted with the embedded ultra-flexible electrode array in the hind paw area of the primary somatosensory (SI) cortex at the following stereotactic coordinates; 1.5 mm rostral and 2.0 mm lateral from Bregma. The rats weighed 222.5 g (*SD* = 3.8 g) at the time of surgery, corresponding to approximately 9–10 weeks. In short, anesthesia was induced by placing the rat in a chamber of 2% isoflurane (Isoba®vet., Apoteksbolaget, Sweden) with 40% oxygen and 60% nitrous oxide (Granmo et al., 2013). The animal was placed on a heating pad and its body temperature was monitored and kept at a stable level. The head was shaved and the rat was mounted in a stereotactic frame (KOPF Instruments, USA). After disinfection of the skin with 70% ethanol, the skull was exposed with a midline incision. The skin was retracted and the skull was cleaned from connective tissue under a stereomicroscope (Leica Microsystems, M651, Germany). Two holes (0.8 mm) were drilled anterior to bregma and one posterior to the SI cortex using a high-speed stereotaxic drill (Dest 300 IN, model MM 323IN, Silfradent, Italy). Small custom-made titanium screws with a length of 1900 μm were screwed ~570 μm into the holes. The choice of material was based on the fact that titanium is a well-known biocompatible material commonly used in implants and which has the potential to integrate into the bone of the skull, yielding increased screw stability and thus adherence of the implant to the skull. The screws were used as anchoring sites for the dental cement (FujuCEM, GC, Europe, Belgium), which was applied at a later stage to attach the electrode/PCB to the skull. The ground wire was wired around the screw and inserted in a separate hole so as to be in contact with the CSF, see below.

A craniotomy, ~2 mm in the rostro-caudal direction, and ~3.5 mm in the medio-lateral dimension was made in the bone of the skull at the SI cortex coordinates, while leaving the underlying dura mater intact. The hind paw area in the S1 cortex was targeted using stereotactical implantation (Kalliomäki et al., 1993). Onset latencies of tactile response were measured from the stimulation artifact. The hole was rinsed with 0.9 % sterile saline solution to clear away any possible debris and prevent tissues from drying.

The embedded electrode was mounted in a hydraulic micromanipulator (KOPF Instruments, USA) and positioned above the SI cortex coordinates. The dura mater was carefully incised and deflected. Care was taken to always keep a thin layer of saline on the cortical surface to prevent it from drying. The electrode was implanted at the above mentioned coordinates to a depth of 1800 μm with a speed of 50 μm/s. After implantation, the ground wire was looped around one of the screws, and subsequently inserted under the bone on top of the dura on the contralateral side of the skull. A small piece of collagen biomatrix for dura regeneration (TissuDura, Baxter International Inc., USA) was positioned in the hole on top of the animal ground wire. The wire was secured and the hole was protected by application of dental cement. To increase the adherence of the PCB and dental cement to the skull, a primer, RelyX™ Unicem, Aplicap™ (3M, Sollentuna; Sweden) was applied prior to dental cement application.

In order to roughly estimate how long after implantation a neural signal could be obtained from the electrodes, a short-lasting recording was made in the anesthetized animal after implantation. In the three animals used for recordings (on 2–4 out of 8 electrode sites in each animal), neuronal activity was detectable already 30–40 min after implantation.

During surgery, the eyes were shielded with a compress which was continually refreshed with physiological saline. In addition, care was taken to make absolutely sure that ethanol or disinfectant was not applied anywhere in the vicinity of the eyes.

After surgery the rat was given subcutaneous injections of 0.01 mg/kg of temgesic (buprenorfin; Schering-Plough, Belgium) to relieve postoperative pain and animals were monitored during the awakening phase. At the end of the experimental period, (3 weeks post implantation), animals were killed by an intraperitoneal injection overdose of pentobarbital and transcardially perfused with saline (0.9%) followed by ice-cold 4% paraformaldehyde (PF) in 0.1 M phosphate buffer, pH 7.4. The brains were post fixed in PF overnight.

### Tissue clarification

To evaluate how well the flexible electrode array maintains its conformation after implantation, the head and brain with the probe still in situ was sectioned and processed according to the clarification protocol described below.

In short, after fixation, the whole head was snap frozen in isopentane on dry ice and stored at −80°C. This preparation method was developed in order to prevent distortion or removal of the electrode from the brain during sectioning of the skull and brain tissue. The top part of the skull bone including the upper part of the electrode was cut off using a circular diamond saw blade mounted on a high-speed dental drill (Desk 200 In, Silfradent, Italy) at 10,000 rpm. After this, the frozen head was cut in 4.5–5.5 mm thick slices with a tissue-slicing machine (ABW 300 GM, 242 W, Avery Berkel, UK), while still kept deep frozen. Care was taken to make sure that the entire electrode array was contained within a single tissue slice (**Figure 3C**).

For clarification the tissue slice was rinsed in 0.1 M phosphate buffered saline (3 × 5 min) to remove the fixative, dehydrated through ethanol of increased concentration (50, 70, and 99.5%, 3–4 h in each), and finally cleared in increasing concentrations (50, 70 in ethanol and 100%) of methyl salicylate (Sigma-Aldrich, Stockholm, Sweden) at least 5 h in each immersion. The conformation of the electrode within the brain slice was examined after clarification and compared with the conformation of the same gelatin embedded electrode before implantation, using 11.5 × magnification stereo microscope (SMZ 1500, Nikon, Japan) with 0.5x WD 136 objective (Nikon, Japan).

### *In-vivo* electrophysiology in awake animals

To evaluate the functionality and stability of the 3D electrode, *in vivo* recordings of single unit activity were made, in awake and non-restrained animals. Neural recordings were performed 2–5 times a week, starting day 1 (1 day after implantation) up to the end point at day 21.

The *in-vivo* recordings were performed in a faraday cage to eliminate external electromagnetic interference. Rats were briefly anesthetized with isoflurane (as per above) to connect the PCB to a 64 channel Plexon data acquisition system via a 20X head stage, a commutator and pre-amplifier (OmniPlex, Plexon Inc, Texas, USA) with a 60 cm long cable. The anesthetic gas was discontinued and signals were recorded from awake and non-restrained rats enclosed in a cage-like container.

To verify the physiological nature of the recorded activity, tactile stimulations of the glabrous skin of the contralateral hind paw were performed by triggering a tactile stimulator connected to a Master 8 stimulator (A.M.P.I., Jerusalem, Israel). At least one session of tactile stimulations was performed per animal. For each session a minimum of 150 tactile stimulations were performed and event time stamps were registered together with the electrophysiological recordings.

### Data analysis

The performance of the developed electrode arrays was evaluated by analyzing the noise levels, signal-to-noise ratio (SNR), yield (ratio of active electrodes that recorded single units and total number of electrodes) and stability of the neural recordings over a period of 3 weeks.

#### Recording parameters

The electrophysiological data were sampled at 16 bit and 40 kHz per channel using a Plexon data acquisition system (Plexon Inc., Texas, USA). The signals were bandpass-filtered (0.25–8 kHz) using a noncausal filter (Quian Quiroga, 2009) and re-referenced using Common Median Referencing (Rolston et al., 2009) to eliminate common-mode artifacts. All post-acquisition analysis was performed automatically using in-house written Matlab (Mathworks Inc.) programs.

#### Unit-identification

Putative single-units were identified using an unsupervised algorithm consisting of a simple amplitude-threshold detector (Quiroga et al., 2004) and clustering with Gaussian Mixture Models (GMM) (Pouzat et al., 2002; Ludwig et al., 2006). The threshold for spike detection was set as minus four times the estimated noise level (standard deviation of background noise) and spikes were aligned temporally at the point of maximum amplitude of the detected valley (Mitra and Bokil, 2008). The noise level was estimated using the maximum absolute deviation (MAD) estimator for standard deviation (Quiroga et al., 2004), given by:

(1) $$\begin{array}{r} {\hat{\sigma }}_{N}=median\frac{\left|signal\right|}{0.6745}. \end{array}$$

As features for spike sorting, the first six principal component analysis (PCA) weights were used (Lewicki, 1998). To estimate the number of units, the feature-distributions were fitted to GMMs with one to six components and the model for which the Bayesian Information Criterion (BIC) converged (BIC < 10% of the BIC-range for all models) was selected. The spikes were assigned to the unit (mixture-component) according to their maximum posterior membership-probability. Spikes that did not have a posterior membership-probability above 0.8 for any unit were classified as outliers (Ludwig et al., 2006).

Signal-to noise ratio (SNR) of units was defined as:

(2) $$\begin{array}{l} SNR=\frac{s_{pp}}{2{\cdot \sigma }_{N}} \end{array}$$

where *s*_*pp*_ is the peak-to-peak amplitude of the unit-waveform and σ_*N*_ is the standard deviation of residuals after subtracting the unit-waveform from each detected spike waveform. ISI (inter-spike interval) violation rate was defined as the percentage of ISIs that were shorter than 1 ms.

An automatic unit-validation procedure largely based on the manual procedure described in Suner et al. (2005) was implemented to identify putative units that could be reasonably assumed to represent neuronal activity. In Suner et al. (2005), this validation was performed by visual inspection of unit-waveforms, and by rejecting units that did not have at least a biphasic component. In order to do this in an objective and consistent manner, we extracted several descriptive features of the unit-waveforms related to this criterion. Those were: (1) polarity of the pre- and post-peak phases of the waveform (identifies strictly monophasic waveforms), (2) the amplitude-difference between waveform endpoints relative to the maximum amplitude (rejecting strongly unbalanced waveforms likely to be artifacts), (3) the number of zero-crossings of pre- and post-peak phases of the waveform (rejecting waveforms with more than 3 zero-crossings in one of the phases) and (4) temporal location of the waveform-peak (rejecting slowly varying waveforms, usually representing artifacts). In addition to these waveshape-related features, we used (5) SNR (rejecting units with SNR < 2 and SNR > 20, representing noise and artifacts, respectively), (6) ISI violation rate (rejecting units with >0.5% ISI violation rate), (7) amplitude-threshold-rate (rejecting units that only marginally crossed the detection threshold), and (8) amplitude (rejecting units with amplitude < 25 uV). The thresholds for the individual metrics were set empirically to obtain consistent rejection of units that would have been likely to be rejected by a manual procedure.

The SNR was further used to classify units as good (SNR ≥ 4), fair (2 ≤ SNR < 4), poor (1 ≤ SNR < 2), and no-signal (SNR < 1). In addition to these classes, we introduced a fifth class containing very-high SNR units (SNR > 6).

#### Recording performance over time

To make a preliminary assessment of the recording performance over time, we measured noise level (Equation 1), SNR of valid units (Equation 2), yield, and relative difference in amplitude between units with highly correlated unit-waveforms recorded on the same channel but on different days. When evaluating performance, the SNR of recordings was taken as the SNR of the highest-SNR valid single-unit identified in the recording. Yield was defined as the percentage of electrode-sites on which at least one valid unit with SNR ≥ 2 (at least fair) or SNR ≥ 4 (at least good) was identified (Ludwig et al., 2006) and was quantified both on a per-week basis and with all days pooled together. To identify units recorded on the same channel but on different days, that might represent the same neuron, we used a method similar to that described in Fraser and Schwartz (2012) in which both the shapes of unit-waveforms and firing statistics were compared between recording sessions. However, since our experiments were performed mainly during spontaneous behavior, no measures related to firing statistics were included in our procedure. Hence, we emphasize that while suggestive, the analysis applied here does not allow safe conclusion on whether or not pairs of valid units with a high correlation between waveform shapes between different recording sessions indeed represent the same neuron.

For all pairs of valid units with SNR ≥ 2 (at least fair) identified on different days, the similarity-measure was taken as the Fisher-transformed maximum value of the cross-correlation between the unit-waveforms. The waveform-correlation threshold for what to consider as units with highly similar waveforms was set as the 90th percentile limit of the distribution of Fisher-transformed waveform-correlations for pairs consisting of units from separate animals. This resulted in a waveform-correlation threshold of 0.96. The correlation threshold was then applied to all same-animal, same-channel, different-day unit-pairs, and pairs with correlation above 0.96 were assumed to putatively represent the same neuron. When multiple unit-pairs were found, only the pair with the highest waveform-correlation was considered.

## Results

### Design features

The resulting electrode array comprised a Z-shaped suspension in the upper part of the shank designed to absorb the major part of the motions between the brain and the skull (Supplementary Video 1). Each of the electrode leads was equipped with a 100 μm long protrusion perpendicular to the main axis, which served a dual function of adding to the flexibility and anchorage in the tissue close to the recording sites. The electrode array was designed to obtain recordings from four different depths, each 400 μm apart, corresponding to layer IV–VI of the rat primary somatosensory cortex. SEM examination of the probe confirmed that a ~100 μm^2^ area of gold surface was de-insulated at the recording sites. To assess the thickness necessary to allow implantation we implanted probes with a thickness of 75 μm (one animal), 100 μm (4 animals), and 125 μm (3 animals). While a thickness of 75–100 μm was deemed to thin to avoid bending during implantation, 125 um thick vehicles did not bend. From these tests we deduced the minimal thickness.

A compression test (Zwick GmbH & Co. KG, Materials Testing Machine with load cell Zwick/Roell KAP-Z (0.04-4N), zero-point deviation 0.02% and pre-load 0.08 N. Acquisition system Zwick/Roell testXpert II) was performed on five gelatin embedded and coated, dummy probes (gold-sheet corresponding to the total area of all 8 leads before individual cut out). The average maximum deformation force (Fmax, force at which the probe begins to bend) was equal to 0.373 N (*SD* = 0.18). This stiffness combined with a needle shaped tip was enough to allow implantation of the electrodes into the tissue with only minor dimpling of the surface.

The shape of the gelatin matrix after molding resulted in a probe with a needle shaped tip and an oval shank diameter (~400 μm wide and ~130 μm thick). To avoid dimpling of the cortical surface during implantation, a sharp tip angle (mean tip angle 41.3°, *SD* = 3.2°) and a tapering distal part is important. The shape used here was therefore a trade-off between the need for stabilization during implantation and a need to keep the size of the probe as small as possible.

### Evaluation of implantation and electrode conformation

**Figure 2:**
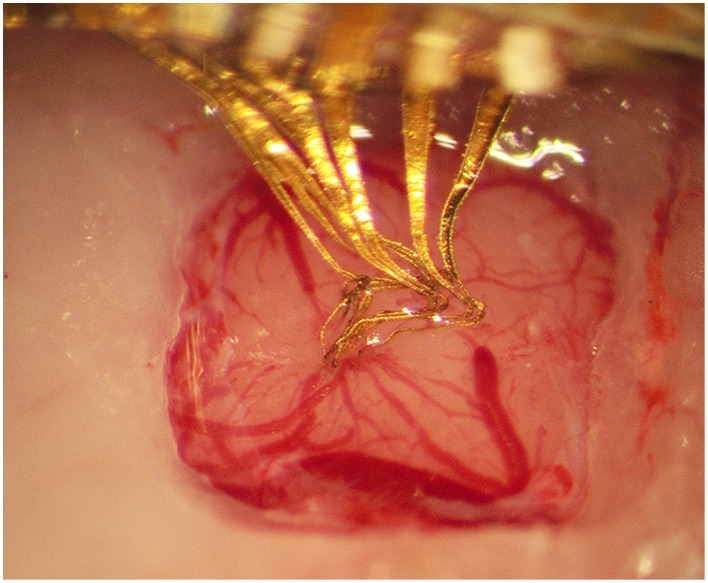
**Photograph taken 3.5 h after implantation of the gelatin embedded electrode, showing that the gelatin has dissolved and the brain surface contracted around the electrode array**.

**Figure 3:**
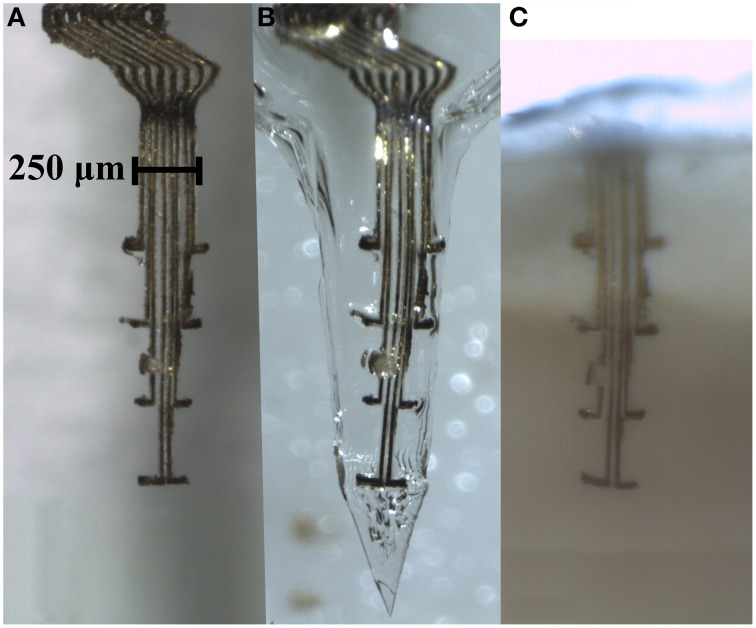
**Electrode array comprising eight thin (4 × 10 μm) gold leads insulated with Parylene C, except for an area (10 × 10 μm) at the tip of the 100 μm long protrusions**. **(A)** An electrode array before embedding into a gelatin based matrix. **(B)** The same electrode array after being embedded into a gelatin matrix shaped as a needle, and in **(C)**, same electrode array inside a section of clarified brain tissue 3 weeks post implantation. Note that the conformation of the electrode array is well preserved inside the gelatin matrix as well as after implantation in the brain.

The implantation into rat cortex proved to be smooth, without bleeding (*n* = 15) or visible conformational changes of the flexible electrode array. The gelatin matrix remained stiff enough to withstand implantation to the intended depth of 1800 μm. To investigate whether any gelatin is likely to remain non-dissolved around the implanted electrode array, gelatin embedded electrode arrays were implanted into the brains of four animals. The brain surface was kept exposed and monitored for 3.5 h. During this time the brain surface was covered by a thin layer of saline to mimic the conditions of the normal implantation procedure in which the skull opening is sealed with dental cement. These experiments showed that the brain surface had contracted around the implant with no visual trace of gelatin in any of the four animals after 3.5 h (Figure 2). Clarification of brain tissue slices with the electrode array still in situ (3 rats) confirmed that the electrode array could be implanted into the brain and remain in the brain for 3 weeks with preserved conformation (Figure 3).

### Quality of recordings in non-restrained awake animals

**Figure 4:**
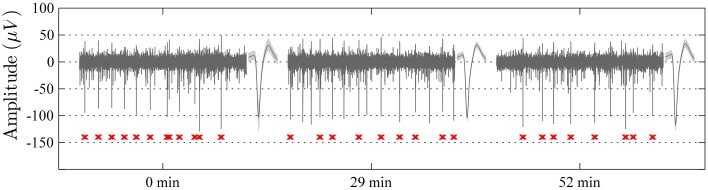
**Samples of 1 s long recordings extracted at 0, 29, and 52 min from one electrode channel in an awake non-restrained rat**. The unit spike with the highest signal to noise ratio in each segment is shown to the right of respective sample (mean waveform ± standard deviation). Note the high level of stability of the recordings with respect to spike-waveforms (waveform correlation > 0.99 between the indicated unit waveforms of all three segments) and amplitude, as well as the overall recording qualities. The spike-times of the indicated single-unit are marked in red below the sweeps.

Evaluation of electrode functionality showed that single unit activity was readily recorded during the full length of the experiment (3 weeks in 3 rats). Single units often remained quite stable both in amplitude and waveform during individual recording sessions (up to 50 min) as shown in Figure 4, indicating that the electrode recording sites remained relatively stable in the tissue during the time-course of individual recordings.

**Figure 5:**
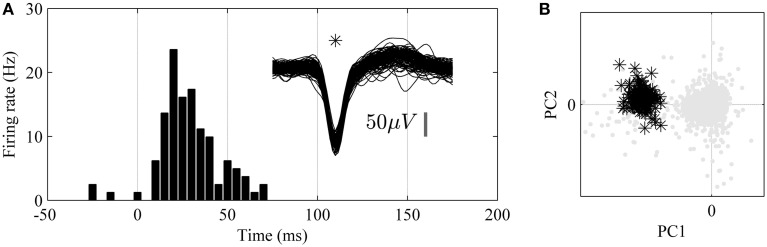
**Analysis of unit responses to tactile stimulation of the hind paw in awake, non-restrained rat**. **(A)** Peristimulus-Time-Histogram (PSTH) for an identified single-unit (superimposed 1.6 ms long spike wave-forms for the unit are shown to the right, *n* = 150). **(B)** Principal component analysis (PCA). Note that the single unit (black stars) is well isolated from the background noise (gray dots) in the principal component (PC1 and PC2) feature space.

In order to verify the physiological nature of the detected spike activity tactile stimulations of the hind paw were performed and correlated to evoked spike activity through the calculation of a peri-stimulus time histogram (PSTH). An example of a single unit with high correlation to tactile stimulation is shown in Figure 5.

**Figure 6:**
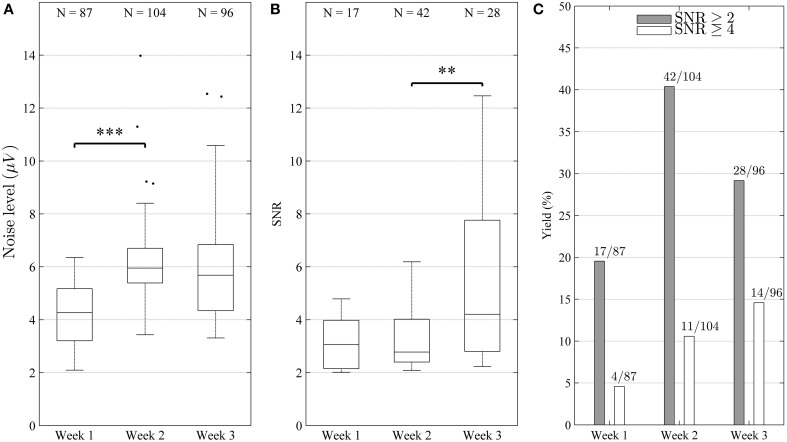
**Characterization of electrode performance over time**. **(A)** Noise level estimated by Equation (1) (distributions shown as median and percentiles values) including all electrode channels. The noise increased significantly (^***^p < 0.001) between weeks 1 and 2, but remained stable between weeks 2 and 3 (*p* > 0.05). **(B)** Signal to noise ratio (SNR) of single-units (distribution including all identified units shown as median and percentile values) remained stable (*p* > 0.05) between week 1 and 2, but increased significantly between week 2 and 3 (^**^p < 0.01). This reflects the fact that high amplitude units were added during week 3. The increase in SNR and yield suggest that the overall recording conditions improved during the course of the experiment. **(C)** Bar diagram depicting yield in percentage (number of channels with units divided by number of channels is shown on top of each bar). It can be seen that yield increased for both good (SNR ≥ 4) and fair (SNR ≥ 2) units **(C)**, although the increase was not strictly monotonic for fair units (peaked during week 2).

The signal quality was good throughout the experiment with an overall median noise level of 5.42 μV (IQR^1^ = 2.16 μV) and overall median single-unit SNR of 3.30 (IQR = 2.30), respectively. As can be seen in Figure 6A, the noise level increased significantly (Mann-Whitney test, *p* < 0.001) between weeks 1 and 2, or from 4.27 to 5.95 μV (median) but remained stable (*p* > 0.05) between weeks 2 and 3. The SNR remained stable (*p* > 0.05) between weeks 1 and 2, but increased significantly (*p* < 0.01) between weeks 2 and 3, from 2.78 to 4.21 (median) (Figure 6B). In some cases, units with very high SNR (SNR > 6) were identified, indicating that the corresponding electrode sites in these cases were located very close to active neurons. In many cases, several single units with lower SNR were identified on the same channel, indicating that signals from more distant or electrically isolated neurons were also recorded.

Out of all electrode sites, 87.5% yielded at least one fair unit (SNR > 2), and 29.2% at least one good unit (SNR > 4) at some point in time. Over the course of the experiment, the weekly yield for both good and fair units showed a clear tendency to increase (Figure 6C).

### On the stability of unit recordings

To assess the positional stability of electrodes within the time-span of individual recording sessions, we measured the variation in mean spike-amplitude for every identified single-unit within a 1-s long moving window applied to the entire recording session. The sliding window was applied in order to diminish the influence of noise on the assessment of amplitude-variation. The amplitude-variation for a given unit was quantified as the standard deviation of relative amplitude deviations across all window positions. This analysis showed that the spike-amplitude remained relatively stable during the time-span of individual recordings, with an overall median standard deviation of 8.65% (IQR = 6.21%) across all identified single-units. Thus, for example, a single-unit with average peak-to-peak amplitude of approximately 130 μV (Figure 4) would be expected to vary around 130 μV by approximately 11.2 μV (8.65% of 130 μV).

**Figure 7:**
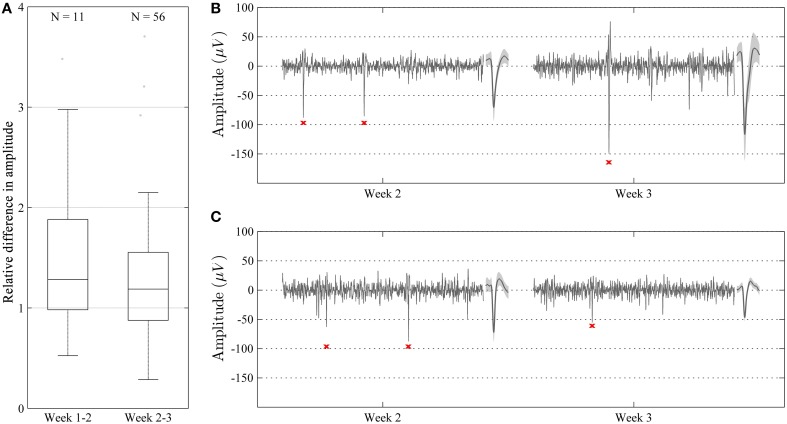
**Characterization of relative amplitudes of single unit spikes with high waveform correlation (>0.96) during two consecutive weeks**. **(A)** Relative difference in amplitude shown as median and percentile values. The amplitude of units showed a tendency to increase over time, but this difference was not significant (*p* > 0.05). **(B,C)** Representative recordings (100 ms long) during week 2 and 3 in two different animals. Recordings are made from the same channel and show highly correlated units (waveform correlation > 0.96). Mean waveform ± standard deviation is shown to the right of each recording. In some cases, the amplitude of the units increased from week 2 to 3 **(B)**, whereas in some other cases, the amplitude decreased **(C)**.

Single-unit recordings obtained during different recording sessions with a waveform correlation higher than 0.96 [see Section Materials and Methods (subsection Recording Performance Over Time)] were obtained during the larger part of the experimental period in all animals. The relative difference in amplitude for such correlated waveforms was close to 1 (median = 1.22, IQR = 0.72) during the whole experiment (Figure 7A). No significant (*p* > 0.05) difference in relative amplitude difference was observed between week 1 and 2, and week 2 and 3. However, the relative difference in amplitude tended to be smaller (closer to 1) between week 2 and 3. Despite this relatively low overall difference in amplitude, we did observe some cases where the amplitude (and thus SNR) differed significantly (up to a factor of around 3) while the waveform remained relatively unchanged (correlation > 0.96) (Figures 7B,C).

In 13 out of 87 recordings where single-units were identified, the units with the highest-SNR had amplitudes greater than 150 μV. The maximum amplitude obtained for a unit was 424 uV. When comparing the waveforms of those units only, and with a stricter fixed waveform-correlation threshold of 0.99, 8 putative same-neuron pairs were identified, extending across a period of 1 day to 1 week, all within the period from day 13 and onward. Given that units with spike amplitudes >150 μV are likely to originate from units that are positioned close to the recordings electrodes (Pettersen and Einevoll, 2008; Thorbergsson et al., 2012), the high waveform correlations between recording sessions in these 8 putative same-neuron pairs is consistent with a high level of positional stability between electrode and neuron. It should be kept in mind, however, that when comparing single-units from discontinuous sessions exhibiting a high correlation of waveform shape (even for cases with correlations >0.99), it cannot be excluded that the recordings derive from different neurons since only spontaneous activity was recorded.

## Discussion

In the present study, we provide a viable solution to the long-standing problem of how to implant electrode arrays, which are too flexible to be implanted without support, with retained conformation during and after implantation. The advantage of this new method is that it allows a tailored architecture of ultra-thin, and therefore more biocompatible electrodes, to be implanted in the brain. In other words, the electrode array can be designed to match the architecture of the tissue. In addition, electrodes individually flexible in all dimensions are expected to reduce the effects of micromotions/microforces which are likely to trigger tissue reactions. Furthermore, the free space between individual leads allows for electrode-tissue integration and prevents the array from creating a significant diffusion barrier. Preliminary tests in 3 animals also demonstrate that high quality recordings from single neurons can indeed by obtained using these electrodes, which is very encouraging.

We chose a rather simple array design with relatively few curved ultrathin leads as our first version. However, it should be noted that the gelatin based vehicle can harbor practically any design of electrode array, including individual leads in the nanometer scales (Witteveen et al., 2010; Suyatin et al., 2013), as well as a larger number of individual channels. The design of the electrode array and hence relative position of recording sites can be adapted to the anatomical structure of various target areas demonstrating its potential for a wide range of applications in neuroscience research and in the clinic (Schouenborg, 2013).

### On the quality and stability of the obtained recordings

The SNR and noise levels found in the present study appear to be similar to data from other current electrodes (Ludwig et al., 2006). A fair comparison of signal quality with the performance of other electrodes would, however, require standardized conditions (Ward et al., 2009). The SNR (Equation 2) was defined on a per-unit basis, using the noise level estimation for each unit individually and not the recording noise-level as defined by Equation (1). The reason for this choice was to ensure compatibility with the SNR-classes defined in Suner et al. (2005). In general, the noise-level estimate in Equation (2) was higher than that of Equation (1). Also, the factor 2 in the denominator of Equation (2) is based on the assumption of bipolar unit-waveforms with positive and negative phases of equal amplitude. However, this was very seldom the case for the units reported here, also suggesting a general underestimation of SNR. Moreover, the generally small variation in spike amplitude within the recording-sessions shows that good recording stability can be obtained in the freely moving animal.

For the majority of electrode sites, single-units were detected at different points in time that, according to the waveform shape criterion (waveform correlation > 0.96), might represent the same neurons. For all three animals, such units were detected over the major part of the observation-period of 3 weeks. Some of those units had high spike amplitudes (>150 μV) indicating that the de-insulated electrode recording sites were located close to the neurons. These findings are consistent with the assumption that good positional stability can be obtained when electrodes flexible in 3D are used. Note, however, that while a high correlation is a necessary criterion for classifying unit pairs as originating from same neurons it is not a sufficient criterion. Since recordings were discontinued between sessions it is still possible that unit pairs with a high correlation and with small changes in amplitudes could originate from different neurons (Fraser and Schwartz, 2012).

The presence of small amplitude fluctuations of unit spikes during the recording sessions suggests that micro-motions, while presumably being significantly reduced by the flexible design of the neural interface, still remain between electrodes and recorded neurons. Moreover, due to the embedding technology used here, it is expected that displacement of neurons relative to the electrodes will occur during the initial period after implantation when the tissue retracts as the gelatin gel is dissolved. Characterization of gelatin dissolution showed that gelatin dissolved and the brain contracted around the implant within 3.5 h. The inevitable loss of cells during the implantation procedure, as well as accompanying glial activation (Biran et al., 2005; Polikov et al., 2005; Leach et al., 2010) will also cause some displacement of nearby neurons, and thus instability in recordings, during the initial week (Ludwig et al., 2006). The findings that the SNR and yield of neurons recorded per channel in fact increased over time may be related to such structural changes in the tissue. This also suggests that longer periods of stable and high quality recordings from the same neurons are within reach. Nevertheless, this remains to be evaluated in long-term studies.

### Gel embedded electrode arrays—comparison with chip-based electrode arrays

In this study, we demonstrate that the overall configuration of the array was well preserved after implantation. This implies there were no significant inherent strain forces in the electrode construction, which could have resulted in non-intended changes in electrode conformation after dissolution of the gelatin. Importantly, the individually separated channels (as in contrast to electrodes embedded into a sheet of polymer) allow for better diffusion inside the tissue and the opportunity for the tissue to integrate with the implant thereby creating a seamless integration of electronics and neural networks.

Gelatin was chosen as the main embedding material due to its biocompatible properties. For example, it is well known that neurons thrive on gelatin in cultures (Abranches et al., 2009) and cells embedded in gelatin have been implanted with no adverse signs reported in humans (Watts et al., 2003; Stover and Watts, 2008). Gelatin is also known to minimize bleeding, presumably due the hemostatic properties of collagen, which is the main constituent of gelatin (Marieb and Hoehn, 2010). There was no visual bleeding during and after any of the implantations of these electrodes (total number of implantations in this study *n* = 15). Thus, the minimal bleeding observed during implantation in this study could be due to the hemostatic properties of collagen.

Moreover, in a previous study in our lab we found that the brain is capable of eliminating implanted pure gelatin needles without forming a permanent scar (Lind et al., 2010). By contrast, a “stab wound” scar was clearly present after 12 weeks when using a non-gelatin embedded needle. More recently, we also found that gelatin coating of flexible probes not only significantly reduces microglial activation but also appears to preserve a normal neuronal density in the area adjacent to the electrode, i.e., a 50 μm wide zone around flexible probes (Köhler et al., 2015). This is in sharp contrast to previous studies reporting a reduction in neuronal density ranging between 20 and 60% in this inner zone (Biran et al., 2005; McConnell et al., 2009; Winslow and Tresco, 2010).

### Limitations and future directions

The development of biocompatible neural interfaces is a very time consuming process, involving multiple cycles of design changes followed by *in vivo* testing. In fact, the present study took several years of developmental work. The present work was focused on evaluating the performance *in vivo* during a relatively short period of time. This short-term study shows that the proposed method makes it possible to manufacture and implant arrays of ultra-flexible, individually separated electrodes whose recording quality is improved after 2 weeks. Given these promising results, long-term studies are a logical next step. Such long-term studies are also needed to compare long-term performance with current neural interfaces and to evaluate several potential improvements of the developed neural interface. For example, the exposed de-insulated tips may be provided with surface enlargements to reduce the impedance and thereby the noise level, e.g., by electroplating procedures (Desai et al., 2010; Ludwig et al., 2011; Ejserholm et al., 2014). Also, the flexibility, and thereby presumably stability, can be further improved by e.g., using thinner leads and/or more curvy shapes of the leads. However, thinner electrodes are more prone to break during manufacturing and implantation procedures and this may thus limit the feasibility of such attempts. Notably, the previous assumption that the size of the electrodes *per se* is critical for long-term glial reactions (Stice et al., 2007; Thelin et al., 2011) may however be erroneous since also large implanted probes elicit very small chronic reactions provided they are truly floating in the brain, i.e., that their specific weight is close to that of the tissue (Lind et al., 2013).

While the present results are encouraging, future long-term studies of probe functions would benefit from being performed during well-defined behavioral tasks to permit an analysis of possible probe induced alterations in neuronal activity. Moreover, more frequent recording sessions would make the unit-tracking procedure more reliable due to the possibility to include measures related to firing-characteristics (Fraser and Schwartz, 2012) and facilitate a more precise quantification of the day-to-day changes of the relative position of the electrode.

It should also be noted that, while a primary purpose of embedding the electrodes in a hard but dissolvable matrix material was to provide the necessary support for fragile electrodes during implantation, the possibility of adding drugs to the embedding material could potentially also provide new opportunities in e.g., pharmacology (Ek et al., 2010). Moreover, by incorporating drugs in the embedding matrix material, e.g., neurotrophic factors, which are released locally in the brain during and after the implantation, it may be possible to promote restitution of the neuronal circuits after the injury caused by the implantation (Gámez et al., 2004; Chang, 2009).

## Concluding remarks

While this first design of gelatin embedded electrode arrays most likely can be improved as discussed above, the present results are encouraging since they suggests that stable long-term communications with the same neurons are indeed attainable. From a research perspective, the importance of stable communication lies in that it will open up for an extraordinarily detailed analysis of how the neuronal circuits in the brain functions under physiological and pathological conditions (Schouenborg, 2011). In particular, the identification and analysis of plastic long-term alterations in neural networks, e.g., during learning or during neurodegenerative diseases, would benefit considerably by stable long-term recordings from the same neurons.

From a clinical perspective, stable recordings of neural activity is a prerequisite for developing robust and interactive protocols for enhancing efficacy and safety of stimulation based treatment (for example deep brain stimulation). In addition, the finding that the configuration of the highly flexible electrode array was retained inside the cortex cerebri after 3 weeks, indicate that a seamless integration of electronics and neural circuits is possible in the near future. Such integration, in particular if stability can be achieved over long periods of time, has the potential of enabling highly interesting neuroengineering development in the future, aiming at, for example, restitution of neural circuits after injury.

### Conflict of interest statement

Jens Schouenborg is an inventor of issued patents or patent applications on flexible electrodes embedded in dissolvable matrix materials and cofounder of Neuronano AB that owns the patents. Jens Schouenborg did not take part in the actual data handling. The other authors have no competing financial interests. There has been no significant financial support for this work that could have influenced its outcome.

## Footnotes

^1^IQR = Interquartile-range.
